# White light emitting molecular logic gates

**DOI:** 10.1002/smo2.70063

**Published:** 2026-06-17

**Authors:** Davide Benedetto Tiz, Massimo Catania, David C. Magri

**Affiliations:** ^1^ Department of Chemistry Faculty of Science University of Malta Msida Malta

**Keywords:** charge transfer, electron transfer, fluorescence, intramolecular proton transfer, molecular logic gates, white light

## Abstract

White light emitting (WLE) materials are of considerable interest with commercial and societal applications in video displays and lighting devices. A characteristic property of WLE materials is the luminescence profile spanning from 380 to 700 nm. The design of molecules, in particular, that emit white light is not so straightforward due to Kasha's rule. However, in recent years, significant progress has been made in the understanding of new photophysical mechanisms. Photochemical concepts such as excited state intramolecular proton transfer (ESIPT), twisted internal charge transfer (TICT), vibration‐induced emission (VIE) and aggregation‐induced emission (AIE) are among the designs in the field. Sensory molecules emitting white light remain uncommon, while those with logic‐based capabilities for sensing two or more analytes or physicochemical properties, are even rarer. These latter gems are the focus of this review. Some fundamental concepts of Boolean logic are introduced for the non‐expert, followed by a survey of molecular sensors and molecular logic gates that emit white light. Our aim is to introduce the reader to logic‐based molecules for addressing societal challenges.

## Graphical abstract

White light emitting smart molecules with Boolean logic computing ability are surveyed.

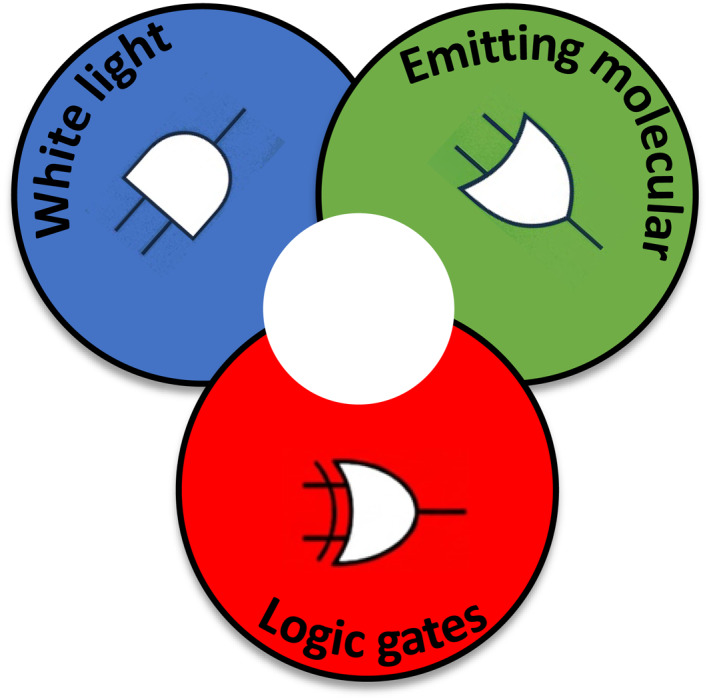

## 1 INTRODUCTION

White light emitting (WLE) materials are of considerable interest with many commercial and societal applications. Of note are the applications in video displays and lighting devices for industrial thin‐film fabrication.[^1^, ^2^, ^3^, ^4^] Government bodies have introduced legislation favorable towards the development of such materials in lighting applications. These initiatives are directed towards reducing energy consumption, carbon footprint and carbon dioxide emissions. A defining characteristic feature of WLE molecules and materials is an emission profile spanning 380–700 nm.[^5^, ^6^, ^7^, ^8^] Single molecules able to generate white light are industrially advantageous by simplifying the fabrication process, and by improving the reproducibility and stability of luminescent solid‐state devices.^[9]^ WLE molecular devices also have potential application in biological contexts as fluorescent probes and biomarkers where semiconductor devices are not compatible. White light emissive probes can improve visibility and contrast in cell imaging applications, allowing for better detection of features in samples. They also provide for tuneable color excitation, which enhances the analysis of stained cell samples. Furthermore, they hold great promise in the field of intelligent optical materials for information processing and transmission.

Designing molecules emitting white light is not straightforward. Unlike the many brilliant colors emitted by specific fluorescent molecules, WLE systems traditionally relied on several colored components to collectively produce white light.[^10^, ^11^] A common approach for some time was to assemble three emitters that simultaneously radiate red, green and blue intensities in nearly equal proportions across the entire visible spectrum.^[12]^ A simpler, alternative approach is to have two complementary emitters that emit orange and blue light.^[13]^ Readers already attuned to Boolean algebra will recognize a cooperative need, that is, of an AND logic mindset in the pursuit of white light generation.

However, the generation of white light is not as easy as mixing three fluorophores emitting blue, green and red in a solution or linked to the same molecular backbone. A common reason is Förster resonance energy transfer (FRET).^[14]^ Spectral overlap of absorption and emission spectra between fluorophores causes the emission to cascade from a higher energy to a lower energy fluorophore. Alternatively, a through bond energy transfer (TBET) mechanism in covalently attached donor‐π‐acceptor frameworks often yields a similar outcome.^[15]^ This tendency is consistent with Kasha's rule − emission results from the lowest vibrational band.^[16]^ Nonetheless, WLE molecules could provide unique advantages not possible with single‐colored luminescent molecules. To reiterate, these could include improved visual readability, built‐in ratiometric calibration for cellular imaging studies and multi‐channel outputs. These advantages will be highlighted in the coming discussion.

The objective of this review is to introduce the reader to logic‐based molecules emitting white light for addressing societal challenges. A secondary aim is to arouse the curiosity of researchers, particularly younger researchers, to contribute towards the mind‐stimulating field of molecular logic, and the broader topic of information processing with molecules. At the 8th International Conference on Molecular Sensors and Molecular Logic Gates 2025, hosted by ECUST in Shanghai, the bulk of the seminars entailed molecular sensors and functional materials. The topic of molecular logic and computing, the second pillar of the conference, was under‐represented. Encouragingly, however, the foundations for molecular sensors (optical sensors, chemosensors, fluorescent probes)[^17^, ^18^, ^19^, ^20^, ^21^, ^22^, ^23^, ^24^, ^25^] are transferable to the field of molecular logic.[^26^, ^27^, ^28^, ^29^, ^30^, ^31^, ^32^, ^33^, ^34^, ^35^, ^36^, ^37^] Individuals working on sensors for specific analytes or physicochemical parameters are in fact within a subset of molecular logic, a rather exciting interdisciplinary field combining chemistry, mathematics, computer science, physics and biology.[^38^, ^39^, ^40^, ^41^, ^42^, ^43^, ^44^]

Within this review, we illustrate concepts and trends on the rational intelligent design and generation of white light emitting molecules. At times, older texts are cited to note similarities and advances since earlier studies. Readers wishing for more background knowledge on molecular logic‐based computation are referred to the literature cited above.

## 2 AN INTRODUCTION TO MOLECULAR LOGIC GATES

**FIGURE 1:**
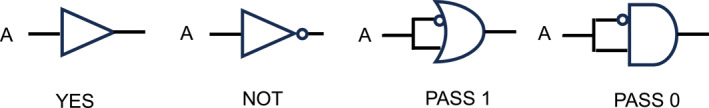
Representative symbols for the four single‐input single‐output logic gates with input A.

Molecular sensors or chemosensors are represented by the classic colorimetric pH indicator introduced in elementary school. Upon accepting ever more protons, the indicator eventually changes color. As a function of pH, a non‐linear S‐shaped profile with plateaux at both ends is observed. The midway point of the titration curve signifies when half of the receptor is bound to protons. In this region of the sigmoidal curve, a small change in the pH stimulus provides a sensitive response output. This is the analog proportion of the curve. The digital aspect is introduced when the plateaux are considered. Thus, a sensor is reconfigured into a single‐input single‐output logic device (Figure 1 & Table 1). The half‐way point is now the threshold level that must be met to turn “on” the indicator. In the field of molecular logic, the output has typically been fluorescence or some other related form of luminescence.^[19]^ The inputs are regularly chemical species, generally inorganic cations and anions, but also biological molecules such as saccharides, amino acids,^[42]^ DNA^[44]^ and enzymes^[43]^ and physicochemical parameters such as temperature, light and polarity.[^35^, ^40^]

**TABLE 1:** The truth tables for the four single‐input single‐output logic gates.

Input	Output			
A	YES	NOT	PASS 1	PASS 0
0	0	1	1	0
1	1	0	1	0

There are four types of single‐input single‐output logic gates (Figure 1). Each gate type YES, NOT, PASS 1 and PASS 0 has two input states and two output states. The classic pH indicator, referred to earlier, with an “off‐on” response is an example of a single‐input single‐output YES gate in Boolean terminology, symbolized by a triangle. When the input A is high (1), the gate communicates a high (1) output, while when the input A is low (0), the gate signals a low (0) output (Table 1). The NOT gate is used to invert an input signal. The symbol for a NOT gate is represented by a triangle followed by a small open circle. When the input A is high (1), the gate converts the output to a low (0), and when the gate signal is low (0), the output flips to a high (1). The PASS 1 and PASS 0 single‐input single‐output logic gates report a steady‐state of high (1) and low (0), respectively, independent of the input condition, whether high (1) or low (0) (Table 1).

**FIGURE 2:**
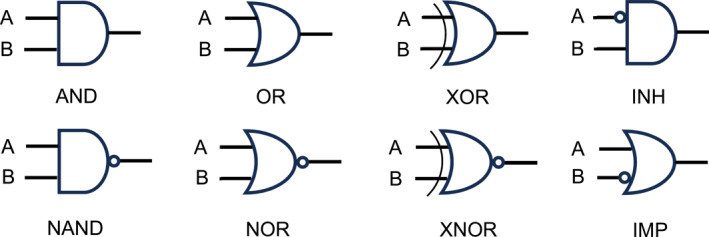
The representative symbols for eight two‐input single‐output logic gates with inputs A and B.

The introduction of a second input exponentially increases the number of logic gate types. There are 16 permutations of two‐input and one‐output logic gates. Figure 2 pictorially highlights the symbols of eight representative two‐input logic gates. The operations most familiar to people in everyday life are the AND gate and the OR gate. The AND gate requires that both inputs A and B be high (1) for a high (1) output to be communicated (Table 2). It is not enough for only input A to be high (1) or only input B to `be high (1). The concept of marriage is a societal example of “two becoming one”. The OR gate is much less demanding − it only requires input A or input B to be high (1) for a high (1) output to be communicated. OR logic is non‐selective. There are three possible input states that give a high (1) output.

**TABLE 2:** The truth tables for eight double‐input single‐output logic gates.

Input		Output							
A	B	AND	NAND	OR	NOR	XOR	XNOR	INH	IMP
0	0	0	1	0	1	0	1	0	1
0	1	0	1	1	0	1	0	1	0
1	0	0	1	1	0	1	0	0	1
1	1	1	0	1	0	0	1	0	1

The NAND function is a combination of an AND gate and a NOT gate. The NOR function is a combination of an OR gate and a NOT gate. NAND and NOR logic gates are examples of combinatorial (also known as combinational) logic gates. The NAND gate is the two‐input version of the NOT gate. When inputs A and B are both high (1), the gate converts the output to a low (0). The NOR gate gives a low (0) output in three cases; only when both inputs are low (0) does the output become high (1).

The INHIBIT (INH) and IMPLICATION (IMP) gates are other examples of combinatorial gates. In these types of gates, a NOT gate operates on one of the inputs (input A in the case of INH, input B in the case of IMP, Figure 2 and Table 2) before transversing the AND and OR components. The INH gate provides a high (1) output only when input A is low (0) and input B is high (1). The IMP gate is the inverse of the INH gate.

Rounding out the two remaining logic gates in Figure 2 are the exclusive OR (XOR) gate, which signals a high (1) output only when input A or input B is high (1). When inputs A and B are both low (0) or both high (1), the output from the XOR gate is low (0). The exclusive NOR (XNOR) gate is an inverted version of the XOR gate. Connections between two or more logic gates lead us towards higher order logic arrays.^[45]^ Now we can begin to do arithmetic. AND and XOR gates in parallel allow for elementary addition, while INH and XOR gates in parallel allow for elementary subtraction.^[26]^ Moreover, we can consider sequential logic, which introduces a memory function or a feedback loop to connect a stored output to a present input. Examples of sequential logic are flip‐flop and keypad circuits.^[40]^

## 3 WHITE LIGHT EMITTING MOLECULES

**FIGURE 3:**
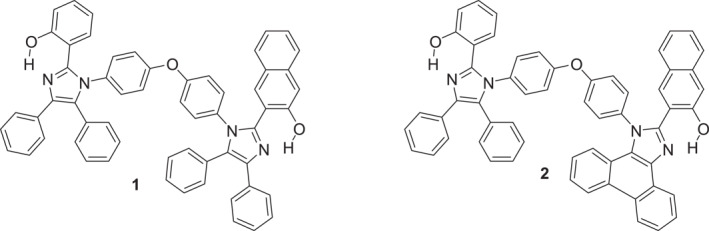
White light emitting molecules **1** and **2** with tetraphenyl imidazole chromophores.

We begin our discussion with examples of WLE organic fluorophores that have made a significant impact on the field of white light generation. Park demonstrated WLE single molecules based on excited‐state intramolecular proton transfer (ESIPT).^[46]^ Two hydroxy‐substituted tetraphenyl imidazole chromophores with phenol and naphthol moieties emit blue and orange. Covalently linking the two chromophores by an ether linkage to yield **1** and **2** provided WLE molecules (Figure 3). White light emerges from the dyad in chloroform and poly(methyl methacrylate) thin films from the superposition of two emission profiles covering the entire UV‐visible region. The fluorescence quantum yields (*Φ*_F_) of **1** and **2** are 0.27 and 0.22, respectively. The design concept prevents concentration dependence effects, and the choice of chromophores prevents spectral overlap and the dissipation of energy from the higher energy chromophore towards the lowest energy pathway. Organic fluorophores have received past criticism for their too often broad emission profiles. In the development of WLE molecules, this broadness is a tremendous advantage. Within the context of Table 1, WLE molecules such as **1** and **2** are examples of PASS 1 logic gates.

**FIGURE 4:**
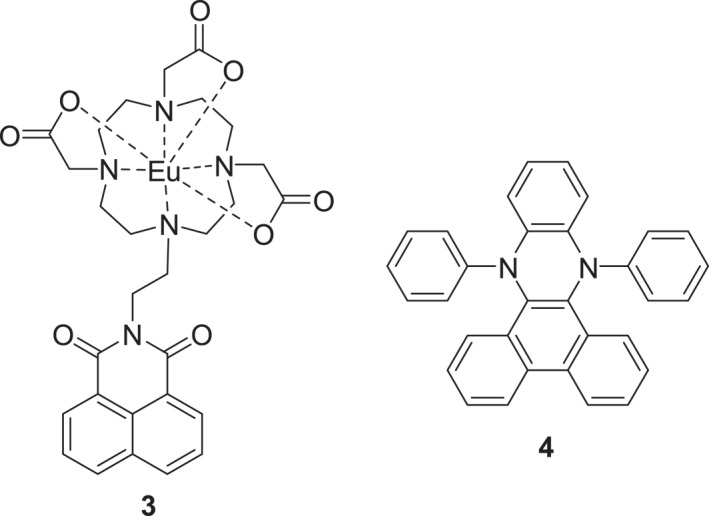
The Eu‐cyclam naphthalimide **3** and dibenzo[a,c]phenazine **4**.

Ward demonstrated a white‐emitting Eu‐complex naphthalimide **3** in 1:1 acetonitrile/water resulting from three phenomena: the naphthalimide monomer emission, the naphthalimide excimer emission and the intrinsic emission of the coordinated europium metal ion within a macrocycle (Figure 4).^[47]^ The blue‐green emission from the naphthalimide chromophore, its excimer emission from aggregation, and the red emission from Eu^3+^ coalesce to give white light. The free ligand emits at 396 nm in aqueous solution. In acetonitrile the emission intensity is reduced, although a second broad band is observed at 475 nm characteristic of excimer emission due to π–π stacking.

Vibration‐induced emission (VIE) occurs in molecules with a configuration that can be altered through a bend‐to‐planar motion and the reverse.^[48]^ The dibenzo[a,c]phenazine **4** has been the prototypical example (Figure 4). The intrinsic change in the planarity of the molecular excited state determines the emission color. Intermolecular interactions do not play a role. When there is planarization along the *N*−*N* axis, there is π‐conjugation throughout the molecule. In the bent geometry, a smaller angle of 138° causes a dramatically lower emission. The amplitude of the emission, dependent on the butterfly‐like motion, is restricted by viscous media. In toluene, a bright white emission with a *Φ*_F_ = 0.22 radiates from a spectrum with maxima at 423 and 613 nm. In the solid state, the phenazine derivative emits a blue emission.

## 4 WHITE LIGHT EMITTING SINGLE‐INPUT LOGIC GATES

Fluorescent molecules with acidic and basic sites have long been used for modulating the emission output.^[20]^ Ideal photoinduced electron transfer (PET) fluorescent sensors traditionally have a fluorophore conveniently distanced from a proton receptor by an alkyl spacer.^[49]^ Fluorescence switching between the “off” and “on” states in PET systems occurs without a change in the emission wavelength. By contrast, fluorescent sensors with acidic and basic sites conjugated directly to the fluorophore with an internal charge transfer (ICT) mechanism are wavelength sensitive to the proton concentration.^[50]^ Protonation (or deprotonation) causes alterations in the electron delocalization of charge. As will be highlighted, complementary colored emissions from two or more proton‐related species in equilibrium may combine to generate white light. Such receptor‐chromophore and receptor‐chromophore‐receptor systems may cause wavelength shifts in the absorbance and emission in an “off‐on” and “on‐off” manner with observable isosbestic and isoemissive points.^[50]^ These constant absorbance and emission wavelengths allow for ratiometric measurements during fluorescence cell imaging studies. In the following section, we proceed with examples of WLE single‐input logic gates.

### 4.1 YES logic

**FIGURE 5:**
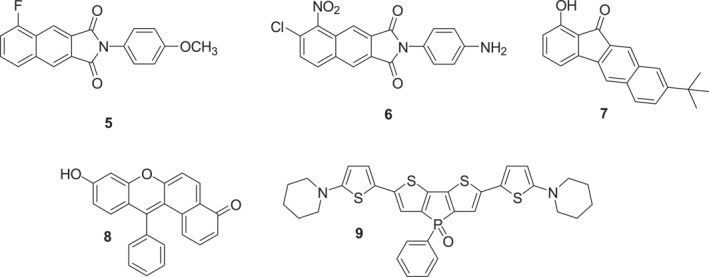
The *N*‐aryl‐2,3‐naphthalimides **5** and **6** have dual emission while the 8‐*tert‐*butyl‐1‐hydroxy‐11*H*‐benzo[b]fluoren‐11‐one **7** has an ESIPT mechanism. Seminaphtho[a]fluorone **8** and dithienophosphole **9** are proton‐responsive white light emitting indicators.

Heagy examined a set of 10 fluorescent *N*‐aryl‐2,3‐naphthalimides.^[51]^ The 5‐fluoronaphthalic amide **5** was selected among the set for use as a ratiometric probe for DNA detection (Figure 5). It has two‐color emission with “off‐on” switching at 428 nm and “on‐off” switching at 506 nm upon interaction with DNA. In aqueous DMSO, excitation at 340 nm generates white light. Compound **6** displays three‐color emission over the entire visible region depending on the excitation wavelength.^[52]^ The emission color is excitation dependent: at 390 nm blue light is emitted while at 450 nm green light is emitted. At 423 nm, the dye emits three‐color peaks that coalesce to give white light (450–700 nm). The origin of these results is believed to come from mixing of π–π* and n–π* excited states.

ESIPT^[53]^ is the mechanism of action in **7** with a hydroxy‐anthracenone–type scaffold (Figure 5). An intramolecular proton transfer reaction at the ketone in the excited state results in a tautomer in equilibrium with the ground state species. Dual emission from both states combine to generate white light.^[54]^ The emission spectrum in cyclohexane has a vibronic structure at 430 and 574 nm. The *Φ*_F_ of the white light is 0.07. Analysis of the geometric structure clearly indicates that the intramolecular hydrogen bond length is shortened upon photoexcitation. By ^1^H NMR an intramolecular hydrogen bond is evidenced by a downfield shift of the proton peak at 8.68 ppm in CDCl_3_.

SNAFR‐1 **8**, a commercially available seminaphtho[a]fluorone molecular probe, can emit white light (Figure 5).^[55]^ Dual emissive probes are sought for measuring analyte concentrations in cells by a ratiometric response. The chromaticity of the emission is a function of both pH and excitation wavelength (270–340 nm). On excitation at 325 nm dual emission is observed in DMSO at 390 and 560 nm. Addition of OH^−^ reveals a ratiometric red emission band for the anionic species at 620 nm at the expense of the green emission at 540 nm for the neutral form. The white light emission was optimized in methanol (*Φ*_F_ = 0.41) with peaks at 385 and 550 nm. Optimally tuned in neutral buffer solution, **8** revealed blue, green and red emission in the right proportions to produce white light. Thus, **8** and other benzannulated xanthene dyes hold promise as pH‐excitation wavelength driven multi‐output logic gates.

A dithienophosphole **9** is designed as a donor‐acceptor molecule capped with piperidine at both ends (Figure 5).^[56]^ Modulation of the acidity generates white light in dichloromethane. In the neutral state, an emission band appears at 657 nm, typical of red light. On protonation with trifluoroacetic acid, green light appears at about 510 nm. Monoprotonation of **9** gives both complementary colored emissions with optimum intensity for producing white light emission.

**FIGURE 6:**
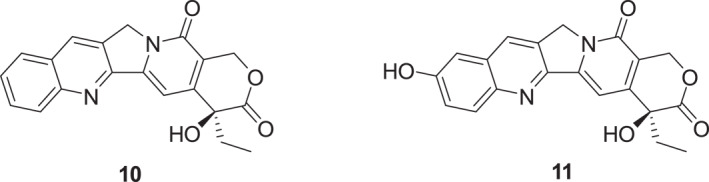
The anticancer drugs camptothecin **10** and 10‐hydroxycamptothecin **11**.

The anticancer drug, camptothecin **10** and its derivative 10‐hydroxycamptothecin **11** (Figure 6) are fluorescent natural products^[31]^ with pH‐dependent WLE properties.^[57]^ In 2:1 ethanol/acetic acid **10**, and in 94:6 ethanol/water and 91:9 ethanol/triethylamine for **11**, WLE is generated from the combined emission of two species, the protonated and unprotonated forms, in equilibrium. At pH 2, the quinoline nitrogen atom is protonated with consequent green emission at 430 nm. At > pH 3 the emission is dominated by a longer band at 540 nm. With isoemissive points at 505 and 520 nm, “off‐on” and “on‐off” switching can be easily interpretable as YES and NOT logic. Incorporated into polyvinyl alcohol films, **11** emits with a *Φ*_F_ of 14% versus 32% in ethanol/water.

**FIGURE 7:**
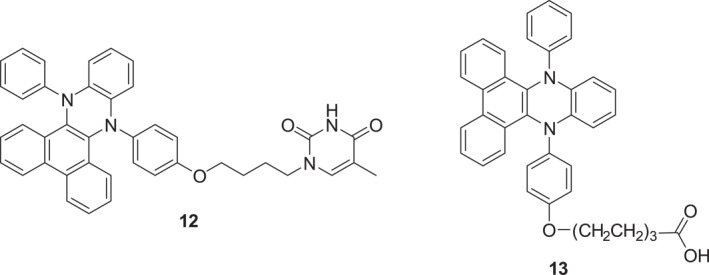
Phenazine‐based molecular probes **12** and **13**.

A phenazine‐based fluorescent probe linked to thymine **12** detects Hg^2+^ ratiometrically (Figure 7).^[58]^ Orange emission is observed in THF/water mixtures up to 76% by volume of water with only slight changes in the emission bands at 470 and 601 nm. Hg^2+^ complexation is indicated by blue emission. The emission increased at 470 nm with a clear “off‐on” emission switching. An isoemissive point about 580 nm is a point of reference. Blue emission comes from a 2:1 **12**/Hg^2+^ V‐shaped aggregate complex. Orange emission indicates a planar coordination 2:1 complex. Pure white light with a chromaticity CIE coordinate at (0.33, 0.33) is witnessed on addition of 2.4 equivalents of Hg^2+^ with a *Φ*_F_ of 11.1%. Addition of sodium sulphide to the solution recovers the orange emission.

Phenazine amphiphiles such as **13** with an aliphatic carboxylic acid tail were used as membrane probes (Figure 7).^[59]^ The mechanism involves unbending in the excited state as opposed to untwisting in the ground state. Red light is observed in ethyl acetate at 592 nm with a *Φ*_F_ of 14.4% from the planar conformation. A weaker blue emission is observed at 465 nm in the bent excited state. In aqueous micelles of dipalmitoylphosphatidylcholine, white light is emitted. The dual emissive property is used to quantitatively measure the extent of micellization. Geometric changes are independent of solvent polarity.[^60^, ^61^]

**FIGURE 8:**
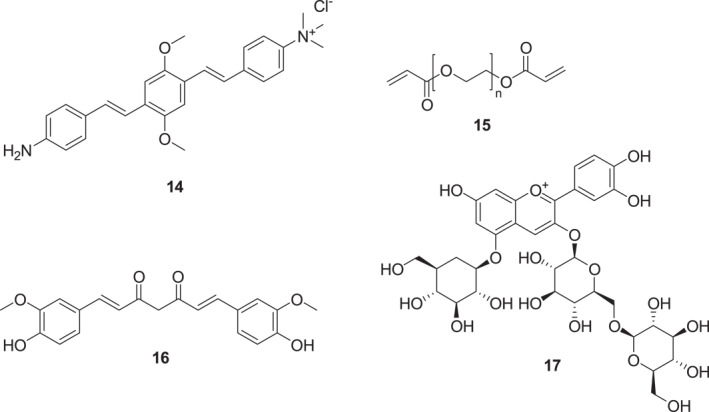
The chemical structures of oligophenylene vinylene oligomer **14**, poly(ethyleneglycol) diacrylate **15** and natural products **16** and **17**.

A trimethylammonium oligophenylene vinylene oligomer **14** has a strong intrinsic dipole moment (Figure 8).^[62]^ The fluorescence spanning from red to blue is associated with a decrease in solution pH. The *Φ*_F_ decreases from 77.8% to 2% at pH 12. The pH‐dependence of the absorption and emission spectra is a superposition of the emission spectra. Two broad peaks at 466 and 600 nm become pronounced with WLE at pH 5. The time‐resolved fluorescence decay lifetimes of 1–3 ns indicate the occurrence of two emission components and the absence of energy transfer.

Next, we have a biocompatible logic system derived from fluorescent natural products.^[63]^ A biodegradable poly(ethyleneglycol) diacrylate **15** hydrogel incorporates the pigments curcumin **16** and cyanidin‐3‐diglucoside‐5‐glucoside **17** (Figure 8). Both pigments have emission profiles covering much of the visible spectrum. Turmeric extract exhibits a wide absorption spectrum from 300 to 500 nm with a peak at 424 nm (ethanol). The anthocyanin maxima of cyanidin‐3‐diglucoside‐5‐glucoside and sinapoyl are identified by high absorption at 283 and 530 nm (1% HCl‐MeOH). On blue excitation, the transparent‐swelling hydrogel detects Hg^2+^ (green) and Cd^2+^ (blue).

**FIGURE 9:**
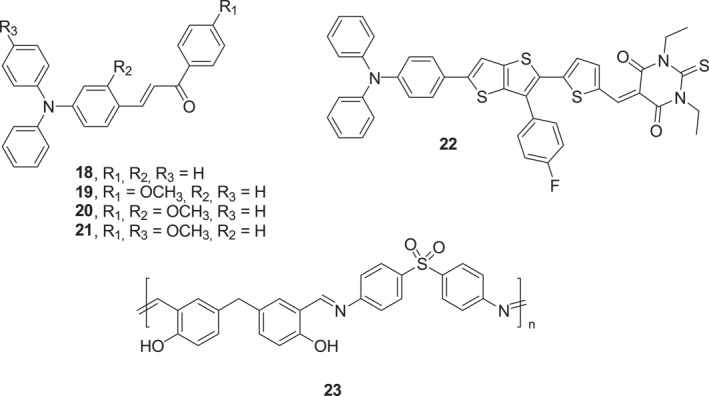
Triphenyl‐chalcone derivatives **18**–**21** and triphenyl‐thiobarbituric probe **22**. The copolymer **23** detects fluoride (F^−^) and cyanide (CN^−^).

Examples **18–21** with a propeller‐shaped triphenylamine are conjugated to a chalcone. They are endowed with solvatofluorochromism (Figure 9).^[64]^ Model **18** is brightly emissive in toluene with a green glow at 510 nm and a quantum yield of 0.32. In CHCl_3_ and DMF, peaks are observed at 566 and 585 nm with yellow emission. Compound **19** with a single methoxy unit experiences a 30 nm blue‐shift and emits white light in DMSO at 443 nm (*Φ*_F_ = 0.02), while **20** with two methoxy units has a greater blue‐shift and white emission in DMSO and EtOH (*Φ*_F_ = 0.02). The white light of **19** and **20** results from the emission of the locally excited state and TICT state in equal proportions. Fluorescence from **21** is only observed in toluene. Strong ICT emission is observed in low polarity solvents while LE emission is uninhibited. In the solid state the emission tends to be yellow or green.

The fluorescent‐probe **22** detects a trio of toxic chemical species,^[65]^ hydrazine, hypochlorite and cyanide in aqueous media (Figure 9). The designed probe has triphenylamine as an electron‐donating group, a fluorobenzene‐thienothiophene moiety as an aromatic spacer, and thiobarbituric acid as an electron‐accepting group. An ICT is associated with the D‐π‐A structure. Reaction with CN^−^, N_2_H_4_ or OCl^−^ generates products with >25‐fold emission enhancements. Identifiable colors are associated with each respective analyte, either white, green or orange light. The emission spectrum of the CN^−^ product extends from 400 to 700 nm, N_2_H_4_ from 440 to 680 nm and OCl^−^ from 450‐700+ nm with peak maxima at 470 nm, 503 and 585 nm, respectively. Nucleophilic addition by the three analytes at the vinylthiophene position results in different fluorimetric products that disrupt the ICT. Cyanide adds to the vinylthiophene, while hydrazine and hypochlorite cleave the thiobarbituric acid unit. A case can be made for categorizing **22** as a two‐input OR logic gate.

Much effort has gone into developing WLE polymers with simultaneous fluorescent and/or phosphorescent emission,[^9^, ^66^] but not so for sensing polymers. A 5,5‐methylene bis(phenol)‐diaminodiphenylsulfone copolymer **23** detects F^−^ and CN^−^ in buffered DMSO solution (Figure 9).^[67]^ Prepared by amine‐aldehyde condensations, an imine moiety connects the two units. Initially colorless and non‐fluorescent, the copolymer detects F^−^ and CN^−^ by an absorbance enhancement at 480 nm with an orange color for F^−^, and 530 nm with a purple color for CN^−^. Bright orange and green emissions with peak maxima at 593 and 492 nm are, respectively, observed. Rather interestingly, the emission from the fluorinated product spans 350–700 nm suggesting that fine tuning of the polymer structure may reveal white light emission. F^−^ is detected by deprotonation of a phenol and CN^−^ is detected by nucleophilic attack at the electron‐deficient carbon atom of the imine group. These reactions induce an ICT mechanism extending over the π‐conjugated orbitals.

### 4.2 NOT logic

**FIGURE 10:**
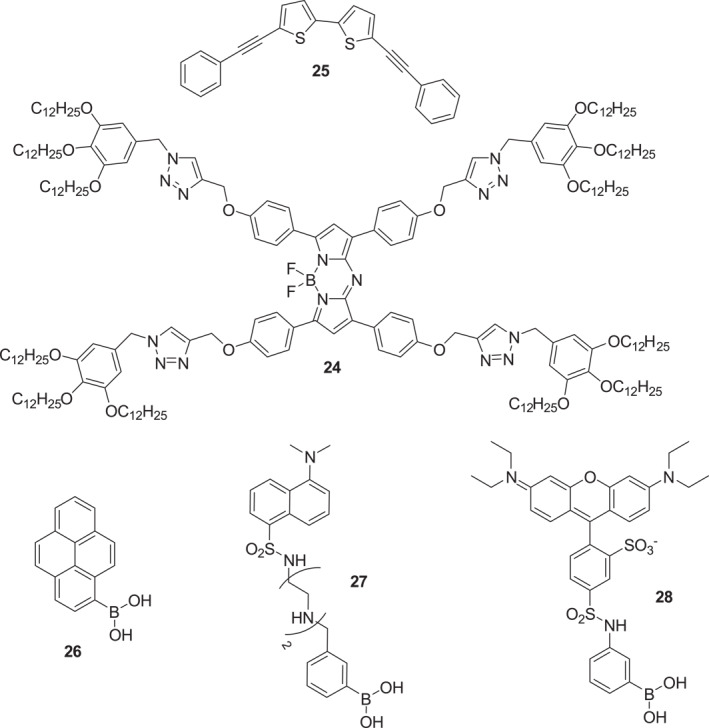
A dendrite‐like aza‐BODIPY chemosensor **24** with blue emitting bithiophene **25**. The tricolor boronic acid dyes **26**, **27** and **28** are surface functionalized to a microparticle.

A dendrite‐like aza‐BODIPY **24** absorbs in the near‐infrared at 680 nm in THF, hexane and DCM, respectively (Figure 10).^[68]^ Emission peaks appear at 730 nm with favorable *Φ*_F_ outputs of ca. 0.38 in THF, DCM, toluene and DMF, respectively. The emission color is dependent on solvent polarity and the aggregation mode (J‐aggregates in the liquid crystalline, gel and solid state). Red monomer emission radiates in dichloromethane. In *n*‐hexane/gel aggregated emission yields a whitish glow. Selectivity occurs with CN^−^ from nucleophilic addition to the aza‐BODIPY mesogen **24** or the intermediate when doped with a blue‐emitting bithiophene amphiphile **25**. Thus, CN^−^ acts as a chemical input to switch “off” the emission consistent with a NOT gate.

Microparticles were surface functionalized with boronic acid‐appended fluorescent dyes of pyrene **26**, dansyldiethylenetriamine **27** and rhodamine B **28** (Figure 10).^[69]^ The tricolor emissive particles exhibit white light emission and function as reusable chemosensors for Cu^2+^ in water. A Cu^2+^‐induced synergistic quenching occurs in aqueous media. Observed with the naked eye, a color change occurs from white to royal purple emission. The decrease in the emission is due to Cu^2+^ complexation with the dansyldiethylenetriamine moiety and from energy transfer from the dansyl to rhodamine B. Selectivity for Cu^2+^ at a detection limit of 15.4 ppb was confirmed against various physiologically and environmentally important metal ions in HEPES buffer. Cu^2+^ switches “off” the white light of this NOT logic gate.

## 5 WHITE LIGHT EMITTING TWO‐INPUT LOGIC GATES

### 5.1 AND logic

**FIGURE 11:**
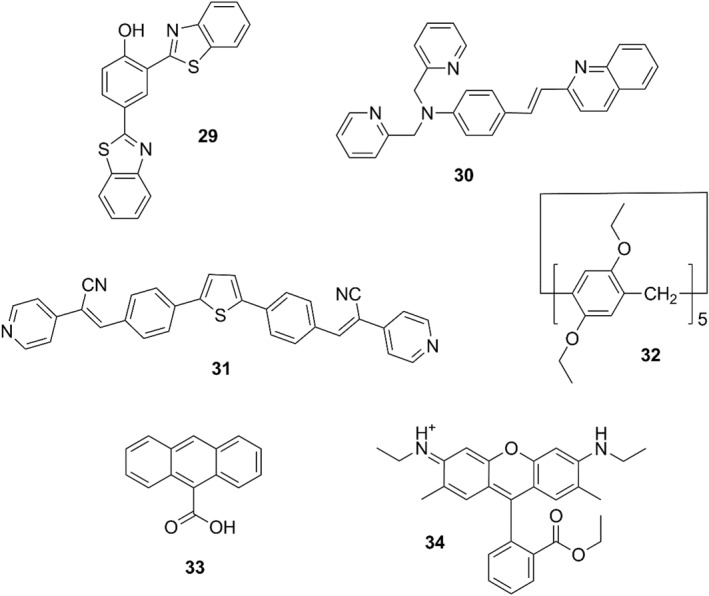
The chemical structures of 2,4‐dibenzothiazolylphenol **29**, dipicolylamine‐quinoline **30**, thiophene α‐cyanostyrene **31**, pillar[5]arene **32**, 9‐anthracenecarboxylic acid **33** and rhodamine 6G **34**.

The emission of 2,4‐dibenzothiazolylphenol **29** is solvent polarity dependent (Figure 11).^[70]^ Blue emission is observed in DMF, while green emission is observed from chloroform. A sharp emissive peak prevails at 485 nm in DMF. An equal ratio of CH_3_Cl/DMF yields white light, consistent with AND logic. In this solvent mixture, the white emission spans 400–650 nm. The white light results from the absorption spectrum of the yellow‐emitting species, which has a small overlap with the fluorescence spectrum of the blue‐emitting species. The minimal overlap is the key factor enabling the simultaneous emission of blue and yellow bands resulting in the perceived white light by an ESIPT mechanism. Perylene and **29** co‐doped in a polymethylmethacrylate film emit white light with a *Φ*_F_ = 0.67.

In contrast, the emission of **30** is dependent on the selection of metal ion.^[71]^ The molecule consists of quinoline connected to dipicolylamine by a conjugated vinyl spacer. The ICT‐based sensor emits a green emission at 508 nm with a *Φ*_F_ = 0.061 in acetonitrile. Blue, yellow, and orange emission are observed upon coordination of Cd^2+^, Hg^2+^ and Pb^2+^ to the dipicolylamine. The emission maxima are 454 nm, 546 nm and 571 nm, and the *Φ*_F_ are 0.033, 0.020 and 0.010. A 1:3 mixture (at 10 times equivalents) of Pb^2+^ and Cd^2+^ provided white light (*Φ*_F_ = 0.014).

White light can be generated by supramolecular assembly. Thiophene‐based α‐cyanostyrene **31** form nanoparticles in THF or DMSO, which is indicated by a green emission (Figure 11).^[72]^ Addition of water significantly lowers the brightness. Host‐guest complexation with the ethoxy pillar[5]arene **32** generates white light. Encapsulation causes the peak at 500 nm to decrease while a new peak at 650 nm appears. At neutral pH the emission spectrum is centered at 520 nm and gradually decreases as the pyridines are protonated. A band at 580 nm indicates the formation of a new assembly morphology. At play is the assembly of the two components by C−H···π interactions within the electron‐rich cavity by the electron‐deficient pyridine groups of the guest, notably when protonated. The cooperative need for the correct solvent conditions, addition of acid and the pillar[5]arene host to generate white light is categorically an AND logic gate.

A composite film consisting of cellulose acetate butyrate and achiral helical polyacetylene acts as a chiral logic gate.^[73]^ The mixing of two dyes, 9‐anthracenecarboxylic acid **33** and rhodamine 6G **34**, emitting blue and orange emission, respectively, achieves white light (Figure 11). Three inputs are considered: chloroform, dichloromethane and 300 nm light. The film is assessed using three techniques, circular dichroism, photoluminescence and chemophotoluminescence, each corresponding to a different output channel. This study brings to mind Schmittel's quadruple‐channel ruthenium‐phenanthroline bis‐aza‐crown ether, which discriminates various metal ions by instrumental technique.^[74]^ Chloroform allows for chirality transfer, while dichloromethane does not due to the higher degree of hydrogen bonding. Hence, we have CHCl_3_‐enabled and CH_2_Cl_2_‐disabled INH logic. Consideration of 300 nm light as a third input results in a chemiluminescence output from a combinatorial INH‐AND logic gate.[^75^, ^76^]

### 5.2 OR logic

**FIGURE 12:**
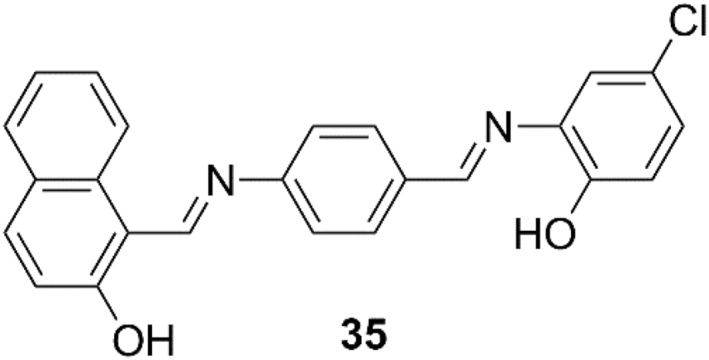
The naphthylidene‐diimine Schiff base **35**.

A naphthylidene‐diimine Schiff base **35** exists as an enol‐imine tautomer in the solid state and a keto‐amine tautomer in DMSO (Figure 12).^[77]^ Yellow, orange and green emission are a visually pleasing sight in solution on irradiation with 365 nm light at neutral, basic and acid conditions. The free ligand emits green fluorescence. The dark yellow solution becomes colorless with Fe^3+^, Cr^3+^ or trifluoroacetic acid and light yellow with Al^3+^ or Cu^2+^ ions. Complexation with Al^3+^ in aqueous DMSO delivers white light with a 12.5‐fold enhancement with maxima at 445 and 539 nm. An ESIPT between the imine nitrogen atom and naphthol OH results in an excited state keto tautomer. The anions OH^−^ and CN^−^ deprotonate the phenol and NH groups within the keto‐amine tautomer to give an orange color.

### 5.3 INH logic

**FIGURE 13:**
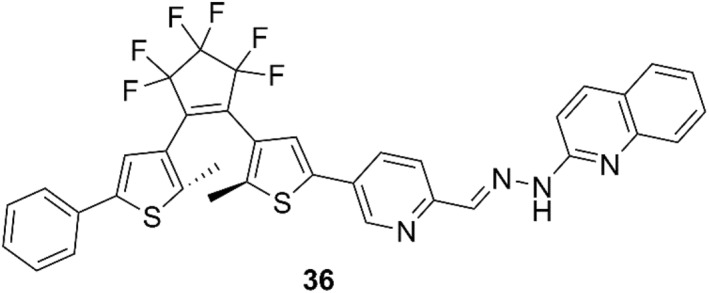
Hydrazinoquinoline **36** is a reconfigurable logic gate with a light‐disabled switch.

An asymmetric photochromic diarylethene with a 2‐hydrazinoquinoline **36** glows in its open form (Figure 13).^[78]^ WLE results from Zn^2+^ complexing with two molecules via the nitrogen‐dense moieties. Cd^2+^ binds by a 1:1 complex with cyan light. Zn^2+^ and Cd^2+^ increase the emission 41‐ and 21‐fold in THF with peak maximum about 510 nm. So, we have a Zn^2+^, Cd^2+^‐driven OR logic in the open form. HSO_4_^−^ emits blue light at 493 nm with a 20‐fold enhancement and a *Φ*_F_ = 0.25. Photocyclization to the closed form with 365 nm light disables the emission in all cases. Irradiation with 500 nm visible light reverses the photocyclization and restores the emission to its original level. Alternatively, **36** is a light, HSO_4_^−^‐driven AND gate when starting in the closed form. Furthermore, the photo switch can be viewed as integrated INH gates connected in parallel with 365 nm light as the disabled input. Photochromic diarylethenes have a history as photochemical light‐stimulated switches.^[79]^ Molecules **45** and **46** are other examples of molecules with an INH function.

### 5.4 IMP logic

**FIGURE 14:**
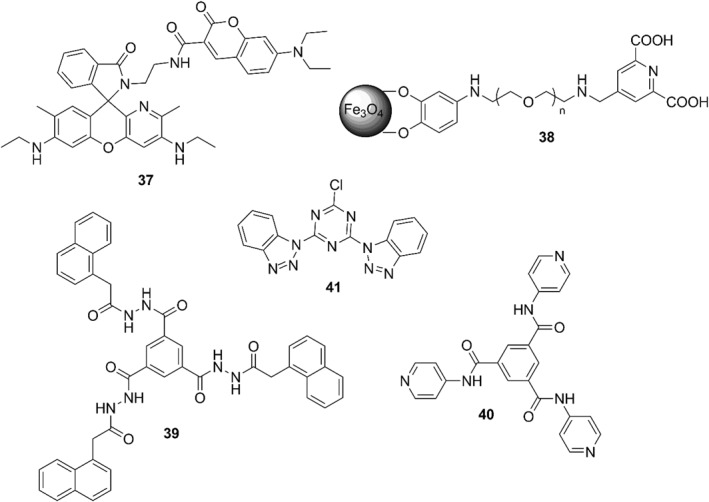
The coumarin‐rhodamine **37** and PEG iron component **38**, tripodal 1‐naphthaleneacetic hydrazide **39**, a tri‐(pyridine‐4‐yl)‐trimesoyl amide **40** gelator and the 1,2,3‐benzotriazole **41**.

A white emissive nanoprobe was demonstrated using a coumarin‐rhodamine‐Eu complex coordinated to dipicolinic acid PEG iron nanoparticles (Figure 14).^[80]^ Eu^3+^ chelates **37** and **38** in a stoichiometry of one iron nanoparticle to four Eu^3+^ and four coumarin‐rhodamine ligands.

The assembly has three emission output channels from coumarin (455 nm), rhodamine (550 nm), and complexed Eu^3+^ (616 nm) in acetonitrile on excitation at 415 nm, 525 and 274 nm, respectively. Blue, green, and red light as well as white emission is observable on excitation at 269 nm. Selectivity occurs for ClO^−^, a bleaching agent and disinfectant, and SCN^−^, a fabric dye and photographic agent. ClO^−^ and SCN^−^ are used as inputs with 616 nm emission as the output. ClO^−^ causes the luminescence to turn off due to ligand exchange. SCN^−^ is innocent to **37** as it does not affect luminescence directly. Rather, SCN^−^ and ClO^−^ react by a redox reaction to generate OSCN^−^ and Cl^−^ with no change in the emission. Described as an INH gate (in negative mode), the nanosystem demonstrates IMP logic.

A bi‐component supramolecular polymer hydrogel forms by hydrogen bonds and π–π stacking interactions between two tripodal gelators, 1‐naphthaleneacetic hydrazide **39** as host and a tripyridine‐triamide **40** as guest (Figure 14).^[81]^ In a DMSO‐H_2_O binary solution, white light is the outcome of 365 nm radiation by aggregation‐induced emission (AIE). The two‐component polymer hydrogel emits fluorescence at 470 nm with a *Φ*_F_ of 0.64. Of many cations screened, the white emission was quenched by Fe^3+^, and of many anions tested, only F^−^ restored the white light. The hydrogel functions as an IMP logic gate only when Fe^3+^ is not present. Temperature is another possible input. As a thin film, the hydrogel is a rewritable fluorescent material with a reading‐erasing‐reading‐writing feedback loop. Heating the gel‐sol to 96°C deactivates the emission and cooling to 25°C restores the white light emission.

Copper nanoparticles were formed from the reduction of copper salts using ascorbic acid and mercaptopropionic acid.^[82]^ Green‐emissive nanoparticles treated for 2 h emit at 520 nm. Blue‐emissive nanoparticles treated for 43 h emit at 440 nm. Duration can be considered as an input condition. Mixed with red‐emitting glutathione capped gold nanocluster in aqueous media white light illuminates. The fluorescence was turned “off” in the presence of Fe^3+^. Association of surface ligands with Fe^3+^ induces the formation of non‐luminescent aggregates. The addition of glutathione reduces Fe^3+^ to Fe^2+^, which restores the luminescence by liberating the metal ions according to IMP logic. White light emerges from mixing the blue and green emissive nanoparticles with orange‐red emitting gold nanoparticles.

Similarly, carbon quantum dots with citric acid and thiourea co‐doped with Cu^2+^ and Mn^2+^ exhibit white light emission with a maximum at 650 nm.^[83]^ Fe^3+^ readily quenches the fluorescence by static quenching in a NOT gate fashion. Ascorbic acid as a second input reduces Fe^3+^ to Fe^2+^, which restores the emission consistent with IMP logic.

A triazine derived from cyanuric chloride and two equivalents of 1,2,3‐benzotriazole **41** emits white light in 24:1 DMF/H_2_O (Figure 14).^[84]^ AIE is the mechanism at work with bluish‐white emission the outcome. Fe^3+^, Cr_2_O_7_^2−^ or nitrobenzene quench the fluorescence most probably from an inner filter effect rather than photoinduced electron transfer.^[49]^ Strong acidic and alkaline solutions both dim the fluorescence. The output is enhanced two‐fold in toluene and xylene. The many input scenarios invite a sequential logic circuit integrating NOT and YES gates reminiscent of a keypad lock.^[85]^ Titled as an *off‐on‐off* system, the classic example of such ternary logic gates uses only one input type.[^86^, ^87^, ^88^]

## 6 WHITE LIGHT EMITTING MULTI‐INPUT LOGIC GATES

**FIGURE 15:**
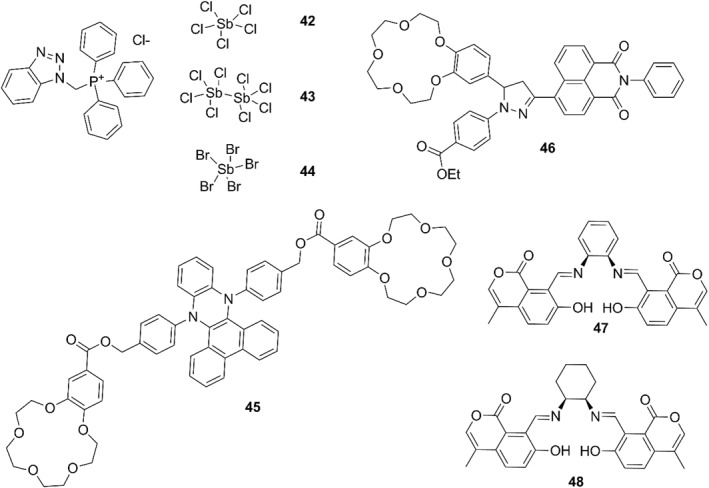
Antimony halide salts **42–44**, benzo‐15‐crown‐5 resettable K^+^‐enabled logic gate **45**, a Na^+^, Mg^2+^‐enabled INH logic gate **46** and coumarin‐salens AND logic gates **47** and **48**.

Benzotriazole triphenylphosphine salts emit various coloured emissions depending on the excitation wavelength. The antimony salt **42** and **43** reversibly switch states in HCl and EtOH (Figure 15).^[89]^ Excited at 300 nm **43** emits pure white light with CIE (0.33, 0.33). Irradiation with a 365 nm lamp reveals orange emission. Excited at wavelengths <300 nm, two peaks are exhibited at 415 and 580 nm. White light emission from the solid state radiates with a *Φ*_F_ of 0.42. Interestingly, **43** is converted to **44** on exposure to HBr. An optical encryption is presented based on a Morse code with a four‐digit code. A temperature and photo‐responsive polymer was demonstrated as a WLE temperature sensor and encryption device based on a FRET mechanism.^[90]^

A phenazine **45** was functionalized with two benzo‐15‐crown‐5 ethers (Figure 15).^[91]^ Orange‐red emission at 605 nm in toluene is associated with a planar emissive geometry. The capture of K^+^ communicates a blue emission at 460 nm due to a change in the conformation and supramolecular assembly. VIE is at play here − a geometry change from planar to bent in the excited state modulates the emission color. Blue emission with 3.5 equivalents of K^+^ signals a K^+^‐driven YES logic gate. White light was observed with 1.5 equivalents of K^+^ in toluene or THF. Dynamic light scattering confirmed that K^+^ causes assembly induced aggregation of a 15‐crown‐5/K^+^/15‐crown‐5 complex. Trace water in the solvent also encourages disassembling and the reemergence of orange emission. The system can now be considered as a K^+^, H_2_O‐driven INH gate. At 460 nm, **45** is a resettable K^+^‐enabled, H_2_O, 15‐crown‐5 disabled NOR‐AND combinatorial gate. This logic behavior contrasts with a bis benzo‐15‐crown‐5‐ether appended to anthracene, which demonstrated intramolecular capture of Cs^+^ besides complexation with two Na^+^ ions as an Cs^+^, Na^+^, H^+^‐driven OR‐AND combinatorial gate.^[92]^

A 3‐pyrazolinyl‐naphthalimide hybrid **46** (Figure 15) recognizes a trio of biologically relevant cations.^[93]^ The benzo‐15‐crown‐5 receptor readily captures Na^+^, but also Mg^2+^ in acetonitrile. UV irradiation activates a red emissive (*Φ*_f_ = 0.095) suggestive of an ineffective PET from the crown ether to the fluorophore. Na^+^ capture modulates the emission color to orange and elevates the brightness (*Φ*_f_ = 0.27). Mg^2+^ capture results in white light and an even brighter output (*Φ*_f_ = 0.40). The third input H^+^ spells trouble, protonating the nitrogen at the 2‐position of the pyrazoline chromophore and deactivating the ICT and consequently the emission output. It functions as two INH gates connected in parallel with H^+^ as the disabling input when excited at 365 nm. But that is not all. The logic gate is a functionally integrated wavelength‐reconfigurable molecular device. Excitation at 470 nm provides a three‐input OR‐INH combinatorial logic gate. The presence of Na^+^ or Mg^2+^ (or both) provides emission while H^+^ turns it off.

As logic gates with Na^+^ and Mg^2+^ inputs are rare, the two coumarin‐salens **47** and **48** are included for discussion (Figure 15) even though they do not emit white light.^[94]^ The presence of both cations is communicated by a bright green fluorescence, but Na^+^ or Mg^2+^ alone leaves the salens non‐emissive. Selectivity for Mg^2+^ is favored in the presence of Na^+^. Weak, broad emission bands appear at 550 and 510 nm. In THF **47** has a 5‐fold‐FE and **48** has a 10‐fold FE. The latter also binds Zn^2+^ with a 39‐fold FE. Na^+^ causes a 36‐fold FE accompanied by an ICT emissive blue shift from 550 to 490 nm. The second probe **48** has a 111‐fold FE and a blue shift from 510 to 430 nm. Na^+^ is postulated to interact with an electron lone pair on the oxygen atom of OH groups (and possibility a carbonyl of a coumarin).^[95]^ Mg^2+^ coordinates to the N2O2 atom set, which reduces the nonradiative decay of the excited state. Coordination of the metal ions to THF may also play a role. The synergistic effect of Na^+^ and Mg^2+^ is consistent with AND logic. The transition metals Zn^2+^ and Cu^2+^ reconfigures the salens to Zn^2+−^enabled and Cu^2+^‐disabled INH logic gates.^[96]^

## 7 CONCLUSION

WLE molecules and materials hold great promise as intelligent devices for societal applications. In this review, we have highlighted a diverse range of design strategies and photophysical mechanisms, including ESIPT, TICT, AIE, VIE, and FRET for achieving white light emission. A summary of the various logic gates, along with key parameters, is provided in Table 3. The reader will notice that a great majority of the examples are YES logic gates (“off‐on” switches), and to a lesser extent, two‐input AND, OR, INH and IMP gates. Other two‐input WLE logic gates, such as NAND, NOR, XOR and XNOR, introduced in Table 2 have yet to be demonstrated. Furthermore, only a few examples of combinatorial logic gates, that is, those integrating two or more two‐input logic gate types, are available. Hence, there are presently gaps to be filled. More sophisticated multi‐functional and reconfigurable logic gate arrays await to be demonstrated. To achieve these more complex arrays, it may be necessary to design molecules with two (or more) of the above‐mentioned photophysical mechanisms. Hence, we are only at the near beginnings of this emerging field intersecting materials science, supramolecular chemistry and molecular logic.

**TABLE 3:** A summary of key performance metrics of representative WLE logic gates.

Molecule	Logic gate	Solvent/media	Inputs	*λ*_em_/nm	Output Φ_F_	Mechanism	Ref.
**1** **2**	PASS 1 PASS 1	CHCl_3_ PMMA	‐ ‐	472 473	0.27 0.22	ESIPT ESIPT	46
**3**	PASS 1	MeCN/H_2_O MeCN H_2_O	‐ ‐ ‐	‐ 475 396	0.022 ‐ ‐	Monomer, excimer, metal complex	47
**4**	PASS 1	Toluene	‐	423, 613	0.22	VIE	48
**5**	YES NOT	DMSO/H_2_O DMSO/H_2_O	*λ*_ex_ = 361 nm *λ*_ex_ = 361 nm	428 506	0.0079 ‐	Dual emission	51
**6**	YES	DMSO/H_2_O	*λ*_ex_ = 423 nm	450	0.46	Dual emission	52
**7**	YES	Cyclohexane	*λ*_ex_ = 414 nm	430, 574	0.07	ESIPT	54
**8**	YES	PO_4_^3‐^ buffer/DMSO	pH	390, 560	0.33	ICT	55
**9**	YES	DCM	pH	510 (H^+^), 657	0.39	ICT	56
**10**	YES	EtOH/AcOH	pH	430, 520 (H^+^)	‐	ICT	57
**11**	YES	94:6 EtOH/H_2_O 91:9 EtOH/NEt_3_ PVA films	pH pH pH	430, 560 ‐ 420, 560	0.32 ‐ 0.14	ICT	57
**12**	YES	THF/H_2_O	Hg^2+^	470, 601	0.11	VIE	58
**13**	YES	Aqueous micelles	*λ*_ex_ = 366 nm	465 (bent) 592 (planar)	0.14	VIE	59
**14**	YES	H_2_O	pH	466, 600	0.78 (pH 2)	ICT	62
**15‐17**	YES	H_2_O	Hg^2+^, Cd^2+^	534 (**16**) 450/580 (**17**)	‐ ‐	ICT	63
**18**	YES	Toluene CH_2_Cl_2_	*λ*_ex_ = 370 nm	510 566	0.32 0.35	ICT	64
**19**	YES	THF, MeCN DMSO	*λ*_ex_ = 370 nm	545, 560 443	0.31 0..02	ICT	64
**20**	YES	DCM EtOH	*λ*_ex_ = 370 nm	529 430, 547	0.17 0.02	ICT	64
**21**	YES	Toluene	*λ*_ex_ = 370 nm	512	0.24	ICT	64
**22**	YES YES YES	Water	CN^−^ N_2_H_4_ OCl^−^	470 503 585	‐ ‐ ‐	ICT	65
**23**	YES	9:1 DMSO/buffer	F^−^ CN^−^	593 492	‐ ‐	ICT	68
**24, 25**	NOT	THF	CN^−^	726	0.38	ICT	69
**26‐28**	NOT	H_2_O	Cu^2+^	515 600	0.52 0.34	EN, complex	70
**29**	AND	Solution Thin film	CH_3_Cl, DMF	490 570	0.30 0.67	ESIPT	71
**30**	AND	Acetonitrile	Cd^2+^, Pb^2+^	454, 571	0.014	ICT	72
**31**	AND	THF/H_2_O	H^+^, **32**	500, 650	0.09	ICT	73
**33, 34**	INH INH INH‐AND	Thin film	CHCl_3_ CH_2_Cl_2_ *λ*_ex_ = 300 nm	412, 509 568, 608	CD PL CPL	Superposition	74
**35**	YES OR	H_2_O/DMSO DMSO	Al^3+^ OH^−^, CN^−^	445, 539 418	‐ ‐	ESIPT	78
**36**	OR INH	THF	Zn^2+^, Cd^2+^ Light, HSO_4_^−^	510	0.35	ICT	79
**37, 38**	IMP	MeCN	ClO^−^,SCN^−^	455, 550, 616	0.07	Metal complex	81
**39, 40**	IMP	DMSO/H_2_O	Fe^3+^, F^−^	470	0.64	AIE	82
**41**	OR‐IMP	DMF/H_2_O	Fe^3+^, Cr_2_O_7_^2−^ NO_2_Ph	350, 427	‐	AIE	85
**42‐44**	YES	Solid state	HCl	415, 580	0.42	Metal complex	90
**45**	YES INH	Toluene or THF	K^+^ K^+^, H_2_O	460	‐	VIE	92
**46**	INH INH OR‐INH	MeCN	Mg^2+^, H^+^ Na^+^, H^+^ Na^+^, Mg^2+^, H^+^	365, 470	0.40 (Mg^2+^) 0.27 (Na^+^)	ICT, PET	94
**47, 48**	AND INH	THF THF	Na^+^, Mg^2+^ Zn^2+^, Cu^2+^	490, 430	‐ ‐	ICT	95

Abbreviations: AIE, aggregation‐induced emission; CD, circular dichroism; CPL, chemophotoluminescence; EN, energy transfer; ESIPT, excited state intramolecular proton transfer; ICT, internal charge transfer; PET, photoinduced electron transfer; PL, photoluminescence; VIE, vibration‐induced emission.

Fluorescent natural products^[31]^ are a potential reservoir of WLE molecular logic gates. Camptothecin **10** and its sister molecule 10‐hydroxycamptothecin **11** are pH and solvent dependent generating white light in ethanol/acetic, ethanol/water and ethanol/triethylamine mixtures.^[57]^ Incorporated into a hydrogel, curcumin **16** and cyanidin‐3‐diglucoside‐5‐glucoside **17** can cooperatively generate white light.^[63]^ These examples exemplify nature‐derived pH‐polarity fluorescent probes that could, with further efforts, be applicable to investigating biological and cellular environments. As many fluorescent natural products are active medicinal agents, there is a potential for WLE molecular theranostics.^[97]^

At present, there is only one commercially available WLE fluorescence probe.^[98]^ The ER‐Tracker Blue‐White DPX kit by Invitrogen is used for live‐cell imaging of the endoplasmic reticulum. It is an environmentally sensitive probe that on excitation at 374 nm illuminates with an emission approximately 430 and 640 nm. It is anticipated that with time other kits with various color combinations, for specific organelles and intracellular analytes, will become commercially available. Such kits could provide fluorescent probes with multi‐color output channels (such as red‐green‐blue combinations) in addition to white light.^[55]^ They will undoubtedly be useful tools for simultaneous and real‐time cell imaging by chemical biologists.

**FIGURE 16:**
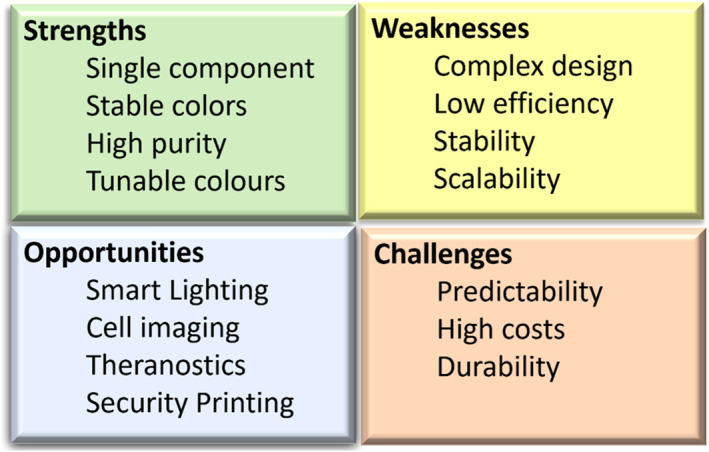
Analysis of white light emitting smart molecules highlighting key strengths, weaknesses, opportunities, and challenges in the context of optoelectronic logic gate applications.

Another societal application of such WLE technology could be for the discrete labeling of object population sets.^[99]^ As with barcodes for cataloging groceries, luminescent logic tags could be used to label proteins and cells or passports and banknotes.^[100]^ Objects tagged with multi‐colored fluorophores, including white light, would exponentially expand the size of the population sets that can be labeled. Tagging of such objects could be used for anti‐counterfeiting measures.^[89]^ Ink‐jet printed room temperature phosphor copolymers could become imbedded as document security features on banknotes, for example,^[101]^ WLE molecular logic gates could be the white knights, so to speak, that solve current and emerging challenges. Within Figure 16 we summarize many strengths, weaknesses, opportunities and challenges. We invite researchers to contribute to this growing niche of molecular logic‐based computation.

## CONFLICT OF INTEREST STATEMENT

The authors declare no conflicts of interest.

## Data Availability

The authors have nothing to report.
